# On Shock Propagation through Double-Bend Ducts by Entropy-Generation-Based Artificial Viscosity Method

**DOI:** 10.3390/e21090837

**Published:** 2019-08-26

**Authors:** Arnab Chaudhuri

**Affiliations:** Department of Civil Engineering and Energy Technology, OsloMet—Oslo Metropolitan University, Pilestredet 35, PB 4, St. Olavs Plass, 0130 Oslo, Norway; arnab.chaudhuri@oslomet.no

**Keywords:** shock diffraction, shock attenuation, entropy generation, high-order numerical scheme, DSEM, artificial viscosity

## Abstract

Shock-wave propagation through obstacles or internal ducts involves complex shock dynamics, shock-wave shear layer interactions and shock-wave boundary layer interactions arising from the associated diffraction phenomenon. This work addresses the applicability and effectiveness of the high-order numerical scheme for such complex viscous compressible flows. An explicit Discontinuous Spectral Element Method (DSEM) equipped with entropy-generation-based artificial viscosity method was used to solve compressible Navier–Stokes system of equations for this purpose. The shock-dynamics and viscous interactions associated with a planar moving shock-wave through a double-bend duct were resolved by two-dimensional numerical simulations. The shock-wave diffraction patterns, the large-scale structures of the shock-wave-turbulence interactions, agree very well with previous experimental findings. For shock-wave Mach number $M_{s}=1.3466$ and reference Reynolds number Ref=106, the predicted pressure signal at the exit section of the duct is in accordance with the literature. The attenuation in terms of overpressure for $M_{s}=1.53$ is found to be ≈0.51. Furthermore, the effect of reference Reynolds number is studied to address the importance of viscous interactions. The shock-shear layer and shock-boundary layer dynamics strongly depend on the Ref while the principal shock-wave patterns are generally independent of Ref.

## 1. Introduction

Shock/blast wave propagation involves complex wave interactions with the media and surface boundaries owing to several phenomena such as shock reflection, shock focusing, shock diffraction and shock-turbulence interaction. Understanding of these phenomena is crucial for a wide range of engineering applications in bio-medicine, disaster management, detonation, mining, aviation/transport industry and others. Knowledge of such complex dynamics is integral part of the design and optimization of devices for shock-wave lithotripsy, shock/blast-wave attenuation, suppression of tunnel sonic boom, etc. The existence of a wide range of flow scales together with unsteady flow discontinuities and the coupled shock-turbulence interactions make it a challenging and active research field. Numerical prediction is a cost effective option compared to its experimental counterpart accounting for several restrictions. However, resolving these unsteady dynamics by numerical techniques demands development of high-fidelity numerical tools. The inspiration behind this work is particularly related to the applicability and effectivity of the high-order numerical tools resolving flow dynamics associated with shock-wave propagation and attenuation.

The literature shows various approaches to attenuate shock-waves, e.g., foams, textiles, porous materials, granular filters, metallic grids, perforated plates/walls, rigid barriers, branched/bend duct, duct with rough walls, etc., as mentioned in [1]. In this regard, a comprehensive review of various methods to attenuate shock/blast waves was reported by Igra et al. [2] addressing both experimental and numerical approaches. Essentially, shock-wave attenuation by geometrical means such as rigid barriers or sudden changes in the flow geometries is governed by compression, rarefaction regions arising from shock diffraction and intense shock-turbulence interactions [1,2,3,4,5,6,7,8,9,10,11]. These involve multiple wave interactions, reflecting from surrounding boundaries and complex shock-shock, shock-vortex and shock-boundary layer interactions. The dynamics may involve transition between regular reflection (RR) and Mach reflection (MR) with triple point. However, depending upon the geometrical complexities and flow conditions, there exists numerous type of irregular reflections [12] and the evolution may consists of complex shock systems with one or more triple point [13,14].

Owing to the enormous advent of computational power and numerical methodologies in recent time, detailed flow dynamics can be retrieved via high-resolution numerical approach compared to experimental measurements. The numerical prediction of complex viscous shocked flow requires stable, high-order numerical schemes to capture shocks as well wide range of flow scales. Often, a competing opposing goal is to be optimized by numerical tools to sustain the accuracy and stability of the scheme. For example, high-order numerical schemes need the addition of dissipation to reduce inherent oscillatory behavior near discontinuities and at the same time one has to reduce numerical dissipation for resolving fine turbulent scales. Successful utilization of explicit high-order Weighted Essentially Non-oscillatory (WENO) has been reported to resolve unsteady shock dynamics related to shock diffraction, shock-reflection and shock focusing [1,5,13,14,15,16,17] problems. On the other hand, high-order Discontinuous Galerkin Methods (DGM) or high-order Discontinuous Spectral Element Methods (DSEM) are comparatively more flexible dealing with complex flow geometries exploiting unstructured mesh-based formulation. Discontinuous spectral element methods (DSEM) can be considered as a nodal DG method [18,19,20,21,22], belonging to the class of weighted residual methods having Dirac delta function as a test function. Spectral element based studies cover a wide range of applications areas related to smooth, non-smooth and complex fluid flow problems [23,24,25,26,27,28,29,30,31,32,33]. Nevertheless, dealing with Gibb’s oscillations for non-smooth fluid flow problems requires implantation of suitable slope limiters or artificial viscosity based approaches. In this regard, Chaudhuri et al. [34] reported the stable shock capturing capabilities of explicit high-order DSEM in conjunction with entropy-generation-based artificial viscosity (AV) method. Although inviscid simulations quite accurately predict the shock wave diffraction wave patterns over different geometrical shapes, it is evident that viscous effects are important for resolving long-time behavior of shock-vortex evolution, shock-shear layer and shock-boundary layer interactions [35,36,37,38,39,40,41]. These issues are also discussed in the recent works [42,43] using high-order DSEM with AV method.

In their works, Igra et al. studied shock propagation and diffraction in ducts with cavity [44,45] and in branched ducts [46]. The effect of attenuation of these configurations compared to uniform cross-section duct is arising from the multiple shock-wave reflections during the propagation of the moving shock. Numerical predictions solving inviscid Euler equations of these configurations agree well with the experimental findings related to general wave patterns and pressure profiles. Subsequently, Biamino et al. investigated the effect of the length of the branched segment revealing the fact that it is questionable to achieve any protection at the end of a branched duct [47]. Nevertheless, favorable shock attenuation by employing abrupt changes in tunnel geometries was reported by Igra et al. [48], highlighting the shock attenuation associated with smooth-walled, rough-walled double-bend ducts. In addition, a detailed experimental findings with respect to varying volume of the double-bend ducts are presented in their work.

The motivation of the present work is to resolve such complex flow dynamics with high-fidelity viscous simulations, which have not been reported in the literature to the best of our knowledge. We aim to present an analysis of shock propagation through the double-bend duct addressing flow behavior with varying reference flow Reynolds number Ref. The $M_{s}$s (shock-wave Mach numbers) are chosen similar to those presented in the experimental work of Igra et al. [48]. A high-order artificial viscosity based DSEM [34] is used for this purpose. The flow analysis sheds light on numerical perspective about the robustness and accuracy of the scheme, as well as the shock diffraction and shock attenuation aspects in double-bend ducts. The paper is organized as follows. A brief description of the governing equations followed by the numerical procedure is given in Section 2. The problem setup is illustrated in the Section 3. The flow analysis and the comparison of the results with the experimental are discussed in Section 4. Finally, conclusions are drawn in Section 5.

## 2. Governing Equations & Numerical Approach

In this section, we briefly present the governing equations and numerical approach. Detail of the numerical procedure is reported in [34] and is not repeated here for brevity.

### 2.1. Governing Equations

Gaseous system involving moving shock-wave is governed by the compressible Navier–Stokes (NS) system of equations. The non-dimensional form of the governing equations with artificial transfer coefficients is given by,

(1) ∂U∂t+∇·Fa(U)−1Ref∇·Fv(U)=0,

and

$$\begin{array}{c} U=\left(\begin{array}{c} \rho \\ \rho v\\ \rho E_{t} \end{array}\right),\;F^{a}\left(U\right)=\left(\begin{array}{c} \rho v\\ \rho vv+p\bar{\bar{\delta }}\\ \left(\rho E_{t}v+p\bar{\bar{\delta }}\cdot v\right) \end{array}\right),\;F^{v}\left(U\right)=\left(\begin{array}{c} 0\\ \bar{\bar{\tau }}\\ \bar{\bar{\tau }}\cdot v+\frac{\kappa_{\mathrm{eff}}}{\left(\gamma -1\right)M_{f}^{2}{\mathrm{Pr}}_{f}}\nabla T \end{array}\right), \end{array}$$

where U is the conservative solution vector, and $F^{a}$ and $F^{v}$ are the inviscid and viscous flux, respectively. ρ is the density, v is the velocity vector, and $E_{t}$ is the total internal energy. *p* is the static pressure and *T* is the temperature. γ is the ratio of specific heats. $\bar{\bar{\delta }}$ is the Kronecker delta tensor. Here, $M_{f}$, Ref and ${\mathrm{Pr}}_{f}$ are the reference Mach number, Reynolds number, and Prandtl number, respectively.

Assuming Stokes’ hypothesis with zero bulk viscosity, the viscous shear stress tensor can be written as $\bar{\bar{\tau }}=2\mu_{\mathrm{eff}}S-\frac{2}{3}\mu_{\mathrm{eff}}\left(\nabla \cdot v\right)\bar{\bar{\delta }}$, where μeff=μhRef+μ is the effective dynamic viscosity and $S=\frac{1}{2}\left[{\left(\nabla v\right)}^{T}+\nabla v\right]$ is the symmetric part of the velocity gradient tensor. The superscript ‘‘T" designates a transpose. Similarly, κeff=κhRef+κ is the effective thermal conductivity. Note that $\mu_{h}$ and $\kappa_{h}$ are yet to be determined artificial transfer coefficients. For NS system of equations, μ=κ=1. In this study, γ=1.4, $M_{f}=1$ and ${\mathrm{Pr}}_{f}=0.72$ were prescribed. The ideal gas equation of state, $p=\rho T/(\gamma M_{f}^{2})$, closes Equation (1).

### 2.2. Numerical Approach

We used the staggered Chebyshev collocation method to approximate the compressible Navier–Stokes system of equations together with the explicit marching algorithm in time. In three-dimensional nodal collocation formulation of DSEM, the physical domain is subdivided with hexahedral physical elements. An iso-parametric transformation is then used to map each physical element to a unit cubic computational element in the computational domain. This reduces to quadrilateral physical elements and square computational elements in the two-dimensional formulation. The solution vector is collocated at the Chebyshev–Gauss quadrature points and the fluxes are collocated at the Chebyshev–Lobatto quadrature points. The detail of the discretization procedure can be found in [19,20,21,22]. Several methodologies can be adopted to suppress the Gibb’s oscillation near the flow discontinuities. For this, a cost effective approach is the usage of AV compared to slope limiters. A short review of different AV approaches in conjunction with high-order methods is reported in [34]. The basic idea of an AV method is to explicitly add even-order dissipation term to stabilize the numerical scheme. Nevertheless, this method requires to define and assign arbitrary model constants as flow-dependent tuning parameters to achieve optimal solution. Additionally, time step restriction arising from artificial viscous terms should also be taken care of. We used entropy-generation-based AV coefficients ($\mu_{h}$ and $\kappa_{h}$) and set a suitable upper bound of these coefficients to judicially address these issues. Here, we present, again, a very brief account of the method of calculation of $\mu_{h}$ and $\kappa_{h}$ to facilitate the understanding the essential features of the numerical procedure.

We consider the non-negative entropy generation terms of the entropy conservation equation in-order to scale the AV coefficients. The artificial momentum and thermal conductivity are scaled with the viscous and conductive, entropy generating terms Φ and Γ, respectively. In non-dimensional form, this yields the following expression for the artificial viscosity coefficients: $\mu_{h}=C_{\mu }\frac{\rho {\left(\Delta h\right)}^{2}}{||\rho \;s-\bar{\rho \;s}{\left|\right|}_{\infty }}\left[\frac{\Phi }{T}\right]$, and $\kappa_{h}=C_{\kappa }\frac{\rho {\left(\Delta h\right)}^{2}}{||\rho \;s-\bar{\rho \;s}{\left|\right|}_{\infty }}\left[\frac{1}{{\mathrm{Pr}}_{f}\left(\gamma -1\right)M_{f}^{2}}\frac{\Gamma }{T}\right]$, where $C_{\mu }$ and $C_{\kappa }$ are model parameters, Δh is the mesh size, $\rho \;s=\frac{\rho }{\gamma \left(\gamma -1\right)M_{f}^{2}}\ln\left(\frac{p}{\rho^{\gamma }}\right)$ and $\bar{\rho \;s}$ is the spatial average of ρs. Here, $||\rho \;s-\bar{\rho \;s}{\left|\right|}_{\infty }$ is the globally computed supremum based on the global average entropy. The artificial viscosity coefficients defined above ensure the positivity (thus dissipative behavior) and $\mu_{h}$ and $\kappa_{h}$ scale with the grid spacing and vanish as Δh→0.

We further used a shock sensor θ [49] to control the artificial viscosity coefficients, so that the modified coefficients can be expressed as $\mu_{h}\theta H\left(-\nabla \cdot v\right),$ and $\kappa_{h}\theta$. This reduces the artificial dissipation in rotation-dominated regions of the flow-field. The purpose of the Heaviside function H(−∇·v) is to ensure that the dissipation is small in regions of isentropic expansion fans and contact discontinuities. The coefficients are kept below an upper bound so that the inviscid time step $\Delta t_{\mathrm{inv}}={\mathrm{CFL}}_{\mathrm{inv}}\Delta h/\left(|v\right|+\sqrt{T}),$ is smaller than the viscous time step. From this constraint, the upper bound of the artificial coefficients becomes $\mu_{h},m=C_{m}\rho \Delta h(|v|+\sqrt{T})$, where $C_{m}\propto {\mathrm{CFL}}_{\mathrm{vis}}/{\mathrm{CFL}}_{\mathrm{inv}}$ represents another model parameter. Note that $\kappa_{h}$ is also kept bounded by the $\mu_{h},m$. Interested readers are suggested to find the detailed description of the overall scheme in [34].

## 3. Problem Setup

**Figure 1:**
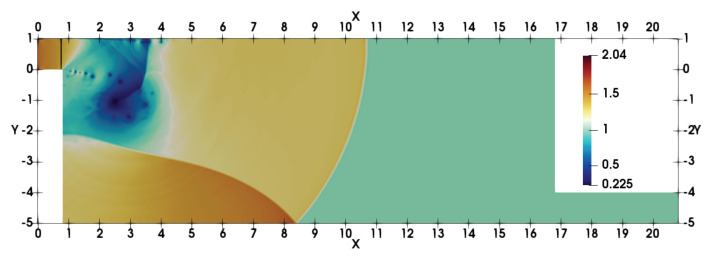
Schematic description of the double-bend duct together with density contours at t=7.2. The black vertical line at x=0.75 shows the initial shock position at the inlet section (top-left). Note that the ratio of length-to-height of the volume inside the double-bend duct is L/H=16.

The geometry of the double-bend duct configuration is taken similar to that presented in the experimental and inviscid study of Igra et al. [48]. Figure 1 shows the setup for the non-dimensional physical domain in the two-dimensional (2D) x−y plane. A shock wave with a suitable Mach number $M_{s}$ is allowed to pass through the duct geometry. The initial shock location is at $x_{s}=0.75$ and the conditions are prescribed using the Rankine–Hugoniot relations for stagnant state (1) and shocked gas state (2). The left and right boundaries are set by the initial states. The top and bottom boundaries are assigned with no-slip wall conditions. The reference state $\phi_{f}$, is taken as the stagnant state conditions $\phi_{1}$, to satisfy $M_{f}=1$ and the reference length is taken as the height of the entrance of the double-bend duct in the experiment [48] $L_{f}=H$. The length of volume inside the double-bend duct is *L* and the chosen geometry satisfies L/H=16 (see Figure 1). Table 1 summarizes the flow properties of stagnant state (1) and shocked gas state (2) for respective values of $M_{s}$.

In total, 68,000 P3 (fourth order) elements with $1.088\times 10^{6}$ degrees of freedom were considered in the domain. In the present study, the value of the CFL number was taken as 0.9 and the model constants for artificial viscosity were set as: ($C_{\mu },C_{\kappa },C_{m})=\left(0.5,0.25,0.15\right)$, unless stated otherwise. Additionally, to enhance the stability of the method, we used adaptive spectral filter with filter order of 16 near the regions where max$\left(\mu_{h},\kappa_{h}\right)$ attains its local upper bound and a filter order of 64 otherwise [34]. The simulations were performed on Supermicro X9DRT compute nodes (dual Intel E5-2670). A typical test case utilized 18 nodes consuming about 3000 CPU hours.

## 4. Results and Discussion

We simulated several cases by setting $M_{s}=1.3466$ and $M_{s}=1.53$ with Ref=103,104,105 and $10^{6}$. Simulations were performed until non-dimensional time t=15. We first discuss the basic flow features for the case ($M_{s}=1.53$, Ref=106) and then subsequently present the comparison for several other cases towards the shock attenuation aspect in double-bend ducts.

### 4.1. General Flow Evolution

**Figure 2:**
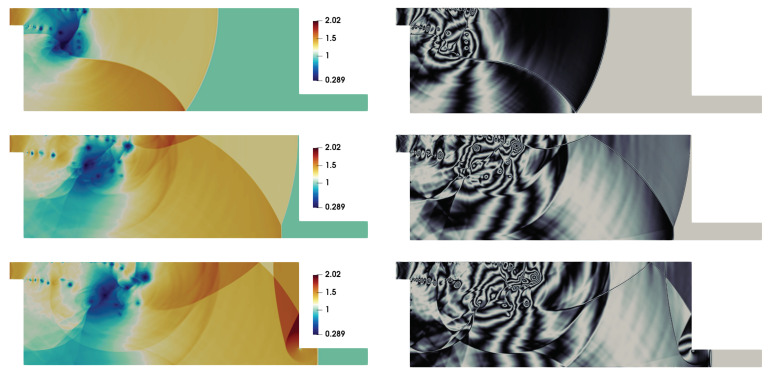
Density contours (left column) and numerical interferograms (right column) at t=8.4,12.4, and 14 (from top to bottom).

**Figure 3:**
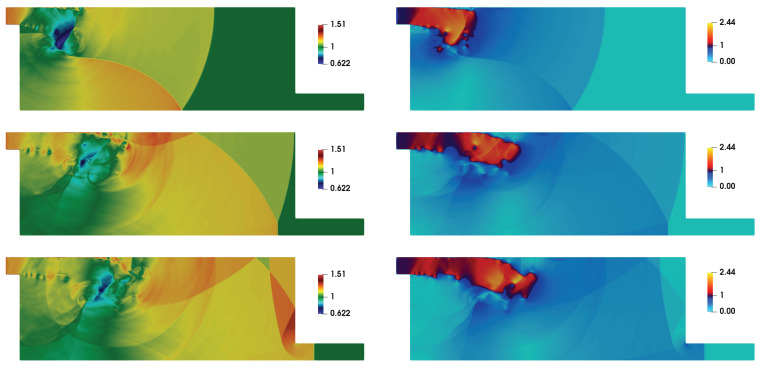
Temperature contours (left column) and Mach number contours (right column) at t=8.4,12.4, and 14 (from top to bottom).

The moving shock-wave after the passage through the inlet section gets diffracted over the top left corner of the domain. It can be realized that the viscous interactions are important to account the shock-wave diffraction over such 90° convex corners. This issue has been discussed with respect to DSEM-AV based numerical scheme in [42]. The flow evolution here, in the double-bend duct, remains similar to that of 90° convex corner until the upward moving reflected shock-wave from the bottom-wall interacts with the diffracted flow field around the corner (see density contour at t=7.2 in Figure 1). In addition, the shear layer interacts with the boundary layer at the top wall through expansion and secondary shock-wave. The density contours in Figure 2 illustrate the subsequent complex flow evolution with multiple transverse wave interactions. The shear layer gets intensely perturbed by the transverse reflected shock-waves, yielding a complex shock-vortex interaction. The unstable shear layer is associated with vortex shedding and vortices become deformed and convected forward in the flow-field. This flow dynamics is also illustrated by the numerical interferograms computed from the density field. The interferograms were computed using the expression: $I=\beta \left[1+\cos\left(2\pi \frac{\rho -\rho_{1}}{\Delta \rho }\right)\right]$. Here, $\Delta \rho =(\rho_{\max}-\rho_{\min})/N$, and *N* is the number of interferential fringes. We chose β=1, as recommended in [50], and N=10 to estimate I. The overall shock-wave patterns, shock-boundary layer interaction, shock-shear layer interactions and the secondary viscous vortex interactions at the left vertical wall interacting at the convex corner are clearly visible in these interferograms. Note that the simulation resolved the RR → MR transition of the shock-wave reflection at the bottom wall (see contours at t=8.4 for RR and at t=12.4 for MR). Contours at t=14 reveal the diffraction at the bottom right corner in the flow domain near the end of the duct. The corresponding temperature and Mach contours are shown in Figure 3. The shocked state of the moving shock is associated the subsonic flow with Mach number is ≈0.63. The red-yellow colored patches in the Mach number contours show the supersonic states in the flow-field at these time instants. The flow again becomes subsonic owing to the secondary shock interaction with the boundary layer at the top surface. The expansion region of the domain acts similar to that of a shock-wave propagation in a nozzle.

### 4.2. Comparison with Experimental Results

**Figure 4:**
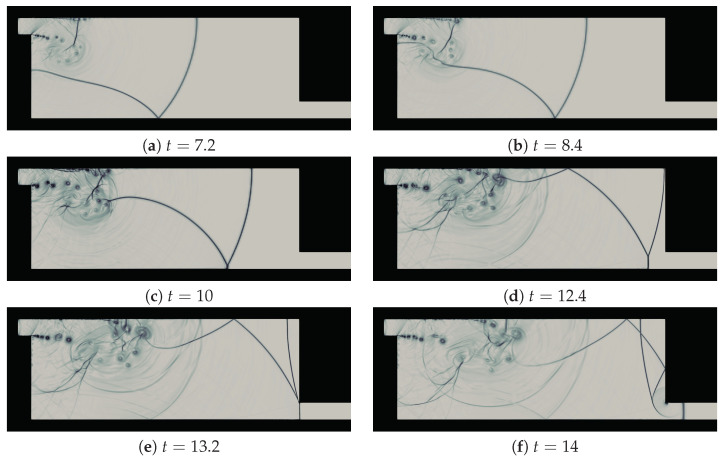
Numerical Schlieren pictures at different time instants.

**Figure 5:**
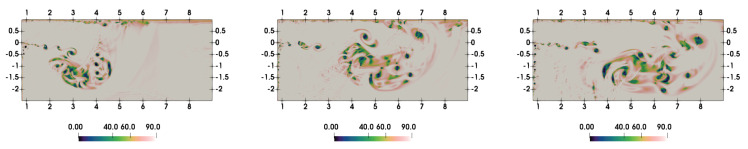
Contours of Ψ for ($M_{s}=1.53$, Ref=106) at t=8.4,12.4, and 14 from left to right.

**Figure 6:**
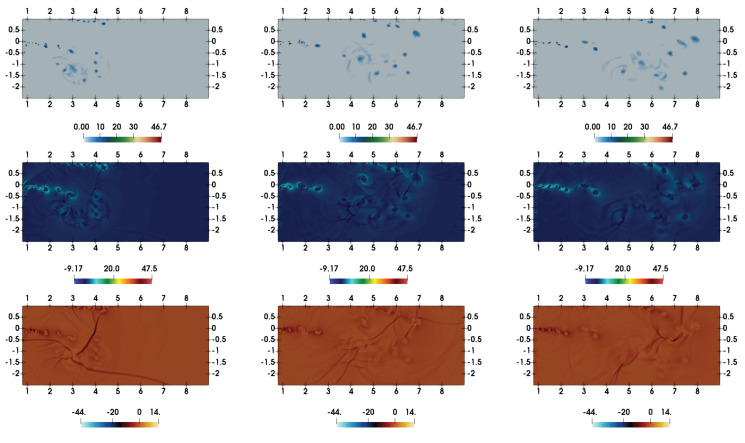
Contours of real and imaginary part of the eigenvalues of the velocity gradient tensor for ($M_{s}=1.53$, Ref=106): (Top row) $\lambda_{i1}$; (middle row) $\lambda_{r1}$; and (bottom row) $\lambda_{r2}$. The contours are at t=8.4,12.4, and 14 from left to right.

We present the time snaps of the flow evolution for the case ($M_{s}=1.53$, Ref=106), to compare with the experimental work of Igra et al. [48]. The numerical Schlieren pictures were computed from the density field using the expression: $S=\beta \exp\left(-\lambda \frac{\left|\nabla \rho \right|}{{\left|\nabla \rho \right|}_{\max}}\right)$. We chose β=1 and λ=15, as recommended in [50], to estimate S. The Schlieren pictures shown in Figure 4 can be compared with the experimental shadowgraphs [48]. The shock-wave patterns from the present simulation are in excellent agreement with the experiment. These resemble to the experimental Shadowgraphs at 220 µs, 260 µs, 300 µs, 380 µs, 400 µs and 420 µs (see Figure 15k,m,o,s,t,u of Igra et al. [48]). The predicted large scale flow structures in rotational dominated regions, and the shear layer dynamics are in very good agreement with the experiment. The global structures of the shock-wave boundary layer interactions at the top wall is also in accordance with the experimental structures. Note that the initial location of the shock-wave for the experimental findings are not known. The flow-field time snaps of the simulation are not exactly at the same instant. The numerical tool equipped with entropy generation based AV together with adaptive spectral filter clearly provides stable and accurate prediction of the flow dynamics. The strain-enstrophy angle can be defined as $\Psi =\tan^{-1}\frac{S\cdot S}{A\cdot A}$. where, $S=\frac{1}{2}\left[{\left(\nabla v\right)}^{T}+\nabla v\right]$ and $A=\frac{1}{2}\left[\nabla v-{\left(\nabla v\right)}^{T}\right]$. The contours of Ψ illustrated in Figure 5 at the interaction zone of window section (0.8≤x≤9,−2.5≤y≤1). The patches of Ψ contours below 45° signify the rotation dominated regions in the domain. For a 2D flow field, the two eigenvalues of the velocity gradient tensor ${\left(\nabla v\right)}^{T}$ can be expressed as $\lambda_{1}=\lambda_{r1}+i\lambda_{i1}$ and $\lambda_{2}=\lambda_{r2}+i\lambda_{i2}$, where $i^{2}=-1$. The instantaneous contours of these eigenvalues are shown in Figure 6 at different time instants. The non-zero high values of $\lambda_{i1}$ are associated with the vortex core regions of shear layer and in the boundary layer regions. From the contours of $\lambda_{r1}$, it can be seen that the high values are adhering to the outer edges of vortices in the shear layer. In addition, in the boundary layer regions, its values are lower where $\lambda_{i1}$ assumes higher values. On the other hand, $\lambda_{r2}$ shows larger values near high dilatation shock regions. These characteristics are similar to that reported in [42]. Nevertheless, it can be realized that three-dimensional (3D) simulations are necessary to further resolve the fine turbulent scales of the flow evolution. An insightful attempt resolving such 3D structures was presented by Chaudhuri et al. [17] via large eddy simulation.

### 4.3. Shock-Wave Attenuation and Effect of Ref

In the review by Igra et al. [2], it is reported that the height of the physical domain is H=60 mm. The experimental condition of the stagnant state is $P_{1}=0.987$ bar, $T_{1}=23.4\;$°C for $M_{s}=1.3466$ and $P_{1}=0.982$ bar, $T_{1}=23.7\;$°C for $M_{s}=1.53$ [48]. These give rise to a reference Reynolds number in the range of $\approx 10^{6}$, based on the length parameter *H* and the speed of sound at stagnant state. We performed simulations for both $M_{s}=1.3466$ and $M_{s}=1.53$ with varying Ref from $10^{3}$ to $10^{6}$. For Ref=103, the model constants for artificial viscosity were set as: ($C_{\mu },C_{\kappa },C_{m})=\left(0.2,0.2,0.1\right)$ and the model constants for remaining cases were assigned to the values mentioned in Section 3.

**Figure 7:**
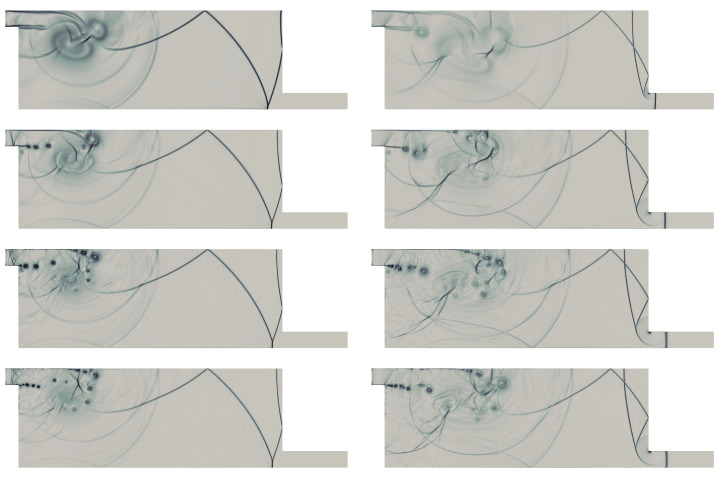
Comparison of numerical Schlieren pictures at t=14 for $M_{s}=1.3466$ (left column) and $M_{s}=1.53$ (right column) with Ref=103,104,105, and $10^{6}$ (from top to bottom).

Figure 7 shows the numerical Schlieren pictures for various test cases at t=14. At this time instant, for $M_{s}=1.3466$, the incident shock-wave reaches near the right bottom corner of the duct while, for $M_{s}=1.53$, it enters the exit section of the domain, after experiencing the second diffraction at this corner of the domain. The basic shock-wave patterns remain unaffected with the variation of Ref. However, the incident shock wave is lagging behind relatively for Ref=103 compared to the higher Refs for both $M_{s}$s. This effect is larger for higher $M_{s}$. For each $M_{s}$, the shock-wave interaction with the main vortex and large scale structures of the shear layer are very much identical for Ref=106 and $10^{5}$. Evidently, dominant viscous effects on the shear layer structures and vortex shedding can be clearly seen for lower Refs. The shear layer becomes stable for Ref=103.

**Figure 8:**
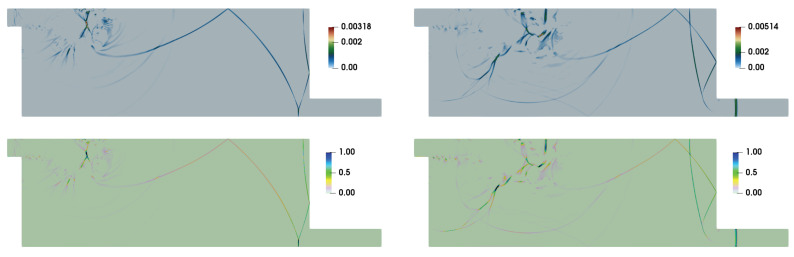
Contours of $\mu_{h}$ (top row) and $\mu_{h}/\mu_{h},m$ (bottom row) at t=14 for Ref=106: (left) $M_{s}=1.3466$; and (right) $M_{s}=1.53$.

**Figure 9:**
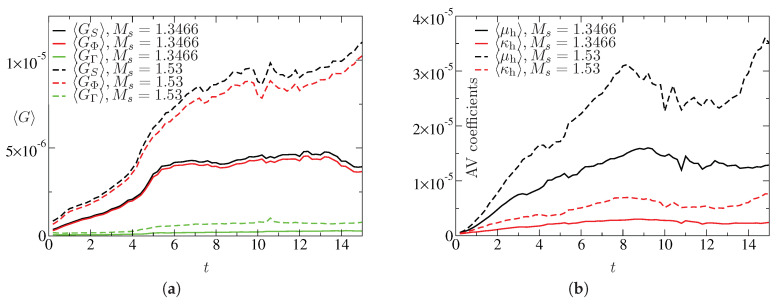
Entropy generation and artificial coefficients for Ref=106: (**a**) evolution of $G_{S}$, $G_{\Phi }$, $G_{\Gamma }$; and (**b**) evolution of $\mu_{h}$ and $\kappa_{h}$.

**Figure 10:**
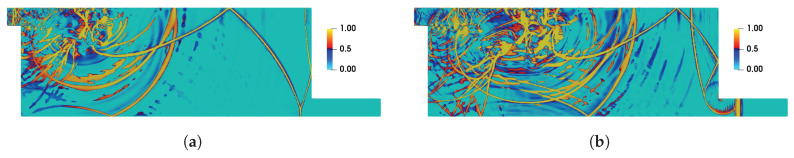
Contours of $G_{S,h}/G_{S,\mathrm{eff}}$ at t=14 for Ref=106: (**a**) $M_{s}=1.3466$; and (**b**) $M_{s}=1.53$.

The entropy generation based AV methodology used in this study is designed to add optimal dissipations to get stable solution. Figure 8 depicts the representative contours of $\mu_{h}$. Note that the intense shock regions of wave patterns are highlighted by the non-zero values of $\mu_{h}$. The contours of the ratio $\mu_{h}/\mu_{h},m$ also show that only few small patches of region are associated with values close to unity. The entropy generation can be expresses as $G_{S}=G_{\Phi }+G_{\Gamma }$, where GΦ=1RefΦT is related to viscous contribution and GΓ=1RefPrf(γ−1)Mf2ΓT is related to thermal contribution (see details of the entropy transport equation in [34]). Figure 9 illustrates the time evolution of area weighted average (defined as $\langle \psi \rangle =\frac{\int \psi dA}{\int dA}$) of these generation terms. This is consistent with the evolution of estimated $\langle \mu_{h}\rangle$ and $\langle \kappa_{h}\rangle$. Evidently, the values of $\langle \mu_{h}\rangle$ remain higher than $\langle \kappa_{h}\rangle$. In addition, note that $C_{\mu }=0.5$, is set higher than $C_{\kappa }=0.25$ for these cases. It is interesting to compare the entropy generation $\left(G_{S,\mathrm{eff}}\right)$, estimated with effective transport coefficients $\mu_{\mathrm{eff}}$, $\kappa_{\mathrm{eff}}$ (see Section 2.1), and the entropy generation $\left(G_{S,h}\right)$, estimated by artificial coefficients μhRef,κhRef. Figure 10 shows the contours of $G_{S,h}/G_{S,\mathrm{eff}}$. It is clear from these contours that the higher values of the ratio are associated with the shock dominated regions and these corroborate with the effectiveness of the shock sensor and the dilatation based Heaviside function for estimating AV coefficients mentioned in Section 2.2.

**Figure 11:**
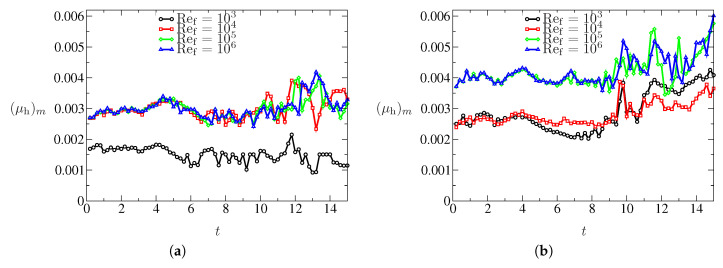
Maximum value of $\mu_{h}$ within the computational domain for various cases: (**a**) for $M_{s}=1.3466$; and (**b**) for $M_{s}=1.53$.

The maximum of $\mu_{h}$ within the computational domain as a function of time is shown in Figure 11 for different cases. Evidently, the maximum values of $\kappa_{h}$ in these cases remain below $\mu_{h}$ (not shown). The range of the values of the AV coefficients lies in a similar range of values found in our previous studies with various applications.

**Figure 12:**
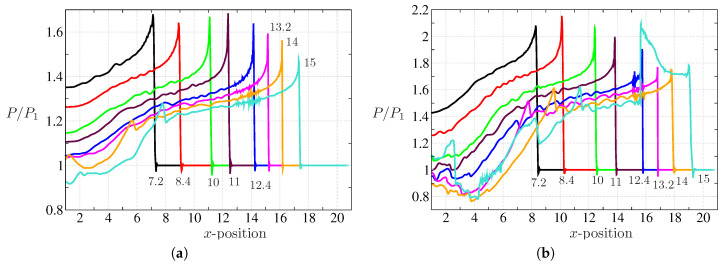
Bottom wall pressure profiles for Ref=106 and (**a**) $M_{s}=1.3466$ and (**b**) $M_{s}=1.53$ at different time instants t=7.2 to t=15, as labeled in the curves.

**Figure 13:**
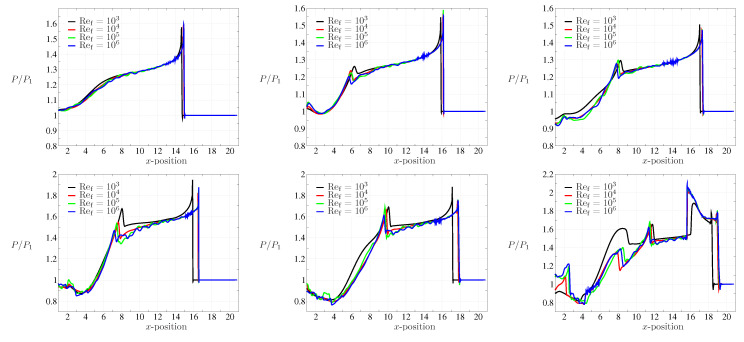
Bottom wall pressure profiles for $M_{s}=1.3466$ (top row), $M_{s}=1.53$ (bottom row) at t=13,14, and 15 (left to right).

We further analyzed the pressure signals at the bottom wall of the double-bend duct for different cases to highlight the attenuation aspect of the flow configuration. The pressure profiles at the bottom wall for both $M_{s}=1.3466$ and 1.53 with Ref=106 are shown in Figure 12. Note that L/H=16 for the double-bend duct considered in this study. For $M_{s}=1.3466$, the value of $P/P_{1}$ predicted from the present simulation near the exit section of the domain is ≈1.5. This value closely matches with the findings of Igra et al. [48] for this configuration. On the other hand, we observed $P/P_{1}\approx 1.8$ for $M_{s}=1.53$. The over pressure can be defined as $\Pi =\frac{P-P_{1}}{P_{2}-P_{1}}$. The outcomes yield the over pressure Π≈0.53 for $M_{s}=1.3466$ and Π≈0.51 for $M_{s}=1.53$. Note that, for $M_{s}=1.53$, the pressure signal also shows the signature of the diffraction from the right bottom corner of the domain. The reflected shock-wave from that corner interacts with bottom boundary and this is clearly seen in Figure 12 at t≈15 with a second peak of $P/P_{1}\approx 2$. In Figure 13, the effect of low Ref is clearly visible. For Ref=103, we observed a shock-wave retardation apart from attenuation. The attenuation features, however, remain unaffected with low Ref when compared with the higher Ref. The transverse wave reflection at upstream locations are also evident in these pressure signals. Note that, there exists an additional hump in pressure signal for the case of $M_{s}=1.3466$. On the other hand, this is similar for $M_{s}=1.53$ at the early stage, while at the later stage, one can notice other humps in the pressure signals (see profiles at t=14,15). Evidently, these are in accordance with the contours presented in Figure 7.

## 5. Conclusions

In this work, we studied the planar shock-wave propagation through a double-bend duct having L/H=16 via numerical simulations. The physics of the flow involves complex shock wave dynamics with shock-wave diffraction and multiple wave interactions together with the shock-shear layer and shock-boundary layer interactions. A high-order DSEM equipped with entropy-generation-based AV method was used to solve Navier–Stokes system of equations for this purpose. The flow evolution for the case ($M_{s}=1.53$, Ref=106) was found to be in excellent agreement with the previous experimental findings of the literature. In addition, for the case (Ms=1.3466, Ref=106), the predicted pressure signal agrees very well with the literature. Additionally, several simulations were performed for two $M_{s}$s with varying reference Ref. The principal shock-wave patterns were found to be generally independent of Ref. On the other hand, the shock-shear layer and shock-boundary layer dynamics strongly depend on the Ref. At Ref=103, we observed marginal retardation of the incident shock-wave, owing to the important role of viscous effects. The results show the applicability and effectiveness of the AV based methodology in resolving complex flow physics associated with the shock propagation and attenuation through double-bend ducts. Three dimensional Detached Eddy Simulation (DES), Delayed Detached Eddy Simulation (DDES) or Large Eddy Simulation (LES) could be performed in the future to resolve later stage fine turbulent flow scales, observed in the previous experiments. 

## Figures and Tables

**Table 1:** Ratio of the flow properties for different $M_{s}$.

$M_{s}$	$P_{2}/P_{1}$	$\rho_{2}/\rho_{1}$	$T_{2}/T_{1}$	$M_{2}$
1.3466	1.949	1.597	1.220	0.456
1.53	2.564	1.913	1.340	0.631

## References

[1] Chaudhuri A., Hadjadj A., Sadot O., Ben-Dor G. (2013). Numerical study of shock-wave mitigation through matrices of solid obstacles. Shock Waves.

[2] Igra O., Falcovitz J., Houas L., Jourdan G. (2013). Review of methods to attenuate shock/blast waves. Prog. Aerosp. Sci..

[3] Tseng T.I., Yang R.J. (2006). Numerical simulation of vorticity production in shock diffraction. AIAA J..

[4] Berger S., Sadot O., Ben-Dor G. (2010). Experimental investigation on the shock-wave load attenuation by geometrical means. Shock Waves.

[5] Chaudhuri A., Hadjadj A., Sadot O., Glazer E. (2012). Computational study of shock-wave interaction with solid obstacles using immersed boundary methods. Int. J. Numer. Methods Eng..

[6] Reeves J.O., Skews B. (2012). Unsteady three-dimensional compressible vortex flows generated during shock wave diffraction. Shock Waves.

[7] Quinn M.K., Kontis K. (2013). Pressure-sensitive paint measurements of transient shock phenomena. Sensors.

[8] Gnani F., Lo K., Zare-Behtash H., Kontis K. (2014). Experimental investigation on shock wave diffraction over sharp and curved splitters. Acta Astronaut..

[9] Gnani F., Lo K.H., Zare-Behtash H., Kontis K. (2015). Shock Wave Diffraction Phenomena around Slotted Splitters. Aerospace.

[10] Wan Q., Eliasson V. (2015). Numerical study of shock wave attenuation in two-dimensional ducts using solid obstacles: How to utilize shock focusing techniques to attenuate shock waves. Aerospace.

[11] Jeon H., Gross J., Estabrook S., Koumlis S., Wan Q., Khanolkar G., Tao X., Mensching D., Lesnick E., Eliasson V. (2015). Shock wave attenuation using foam obstacles: does geometry matter?. Aerospace.

[12] Ben-Dor G., Ben-Dor G. (2007). Shock Wave Reflection Phenomena.

[13] Shadloo M., Hadjadj A., Chaudhuri A. (2014). On the onset of postshock flow instabilities over concave surfaces. Phys. Fluids.

[14] Soni V., Hadjadj A., Chaudhuri A., Ben-Dor G. (2017). Shock-wave reflections over double-concave cylindrical reflectors. J. Fluid Mech..

[15] Chaudhuri A., Hadjadj A., Chinnayya A. (2011). On the use of immersed boundary methods for shock/obstacle interactions. J. Comput. Phys..

[16] Glazer E., Sadot O., Hadjadj A., Chaudhuri A. (2011). Velocity scaling of a shock wave reflected off a circular cylinder. Phys. Rev. E.

[17] Chaudhuri A., Hadjadj A. (2016). Numerical investigations of transient nozzle flow separation. Aerosp. Sci. Technol..

[18] Patera A.T. (1984). A spectral element method for fluid dynamics: laminar flow in a channel expansion. J. Comput. Phys..

[19] Kopriva D.A., Kolias J.H. (1996). A conservative staggered-grid Chebyshev multidomain method for compressible flows. J. Comput. Phys..

[20] Kopriva D.A. (1998). A staggered-grid multidomain spectral method for the compressible Navier–Stokes equations. J. Comput. Phys..

[21] Jacobs G.B., Kopriva D.A., Mashayek F. (2005). Validation study of a multidomain spectral code for simulation of turbulent flows. AIAA J..

[22] Kopriva D.A. (2009). Implementing Spectral Methods for Partial Differential Equations: Algorithms for Scientists and Engineers.

[23] Taylor M., Tribbia J., Iskandarani M. (1997). The spectral element method for the shallow water equations on the sphere. J. Comput. Phys..

[24] Karniadakis G., Sherwin S. (1999). Spectral/hp Element Methods for CFD.

[25] Black K. (1999). A conservative spectral element method for the approximation of compressible fluid flow. Kybernetika.

[26] De Frutos J., Novo J. (2000). A Spectral Element Method for the Navier–Stokes Equations with Improved Accuracy. SIAM J. Numer. Anal..

[27] Taylor M.A., Fournier A. (2010). A compatible and conservative spectral element method on unstructured grids. J. Comput. Phys..

[28] Chauvière C., Owens R.G. (2001). A new spectral element method for the reliable computation of viscoelastic flow. Comput. Methods Appl. Mech. Eng..

[29] Sprague M., Geers T. (2004). A spectral-element method for modelling cavitation in transient fluid–structure interaction. Int. J. Numer. Methods Eng..

[30] Kreeft J., Gerritsma M. (2013). Mixed mimetic spectral element method for Stokes flow: A pointwise divergence-free solution. J. Comput. Phys..

[31] Cantwell C.D., Moxey D., Comerford A., Bolis A., Rocco G., Mengaldo G., De Grazia D., Yakovlev S., Lombard J.E., Ekelschot D. (2015). Nektar++: An open-source spectral/hp element framework. Comput. Phys. Commun..

[32] Ghaffari A., Mustafa I., Javed T. (2018). Time Dependent Convective Non-Orthogonal Hiemenz Flow of Viscoelastic Walter’s B Fluid towards a Non-Uniformly Heated Vertical Surface: Using Spectral Method. Nihon Reoroji Gakkaishi.

[33] Mustafa I., Javed T., Ghaffari A., Khalil H. (2019). Enhancement in heat and mass transfer over a permeable sheet with Newtonian heating effects on nanofluid: Multiple solutions using spectral method and stability analysis. Pramana.

[34] Chaudhuri A., Jacobs G., Don W., Abbassi H., Mashayek F. (2017). Explicit discontinuous spectral element method with entropy generation based artificial viscosity for shocked viscous flows. J. Comput. Phys..

[35] Sun M., Takayama K. (2003). A note on numerical simulation of vortical structures in shock diffraction. Shock Waves.

[36] Sun M., Takayama K. (2003). Vorticity production in shock diffraction. J. Fluid Mech..

[37] Law C., Muritala A., Skews B. (2014). Unsteady flow with separation behind a shock wave diffracting over curved walls. Shock Waves.

[38] Takayama K., Inoue O. (1991). Shock wave diffraction over a 90 degree sharp corner—Posters presented at 18th ISSW. Shock Waves.

[39] Skews B., Law C., Muritala A., Bode S. (2012). Shear layer behavior resulting from shock wave diffraction. Exp. Fluids.

[40] Kleine H., Klioutchnikov I., Olivier H. (2015). Onset of shear layer instability in shock diffraction processes. 29th International Symposium on Shock Waves 2.

[41] Soni V., Chaudhuri A., Brahmi N., Hadjadj A. (2019). Turbulent structures of shock-wave diffraction over 90° convex corner. Phys. Fluids.

[42] Chaudhuri A., Jacobs G. (2019). Dynamics of shock wave diffraction over sharp splitter geometry using entropy-based artificial viscosity method. Shock Waves.

[43] Chaudhuri A. (2018). Shock propagation and diffraction through cavity. Proceedings of the 59th Conference on Simulation and Modelling (SIMS 59).

[44] Igra O., Falcovitz J., Reichenbach H., Heilig W. (1996). Experimental and numerical study of the interaction between a planar shock wave and a square cavity. J. Fluid Mech..

[45] Igra D., Igra O. (2016). Planar Shock-Wave Diffraction into a Square Cavity Filled with Different Gases. AIAA J..

[46] Igra O., Wang L., Falcovitz J., Heilig W. (1998). Shock wave propagation in a branched duct. Shock Waves.

[47] Biamino L., Jourdan G., Igra O., Mariani C., Tosello R., Leriche D., Houas L. (2014). Experimental investigation of shock wave propagation in a 90 degree branched duct. Shock Waves.

[48] Igra O., Wu X., Falcovitz J., Meguro T., Takayama K., Heilig W. (2001). Experimental and theoretical study of shock wave propagation through double-bend ducts. J. Fluid Mech..

[49] Ducros F., Ferrand V., Nicoud F., Weber C., Darracq D., Gacherieu C., Poinsot T. (1999). Large-eddy simulation of the shock/turbulence interaction. J. Comput. Phys..

[50] Hadjadj A., Kudryavtsev A. (2005). Computation and flow visualization in high-speed aerodynamics. J. Turbul..

